# Herpes Simplex Virus Cell Entry Mechanisms: An Update

**DOI:** 10.3389/fcimb.2020.617578

**Published:** 2021-01-18

**Authors:** Krishnaraju Madavaraju, Raghuram Koganti, Ipsita Volety, Tejabhiram Yadavalli, Deepak Shukla

**Affiliations:** ^1^ Shukla Lab, Department of Ophthalmology and Visual Sciences, University of Illinois at Chicago, Chicago, IL, United States; ^2^ Department of Microbiology and Immunology, University of Illinois at Chicago, Chicago, IL, United States

**Keywords:** herpesvirus, HSV, entry, mechanism, endocytosis

## Abstract

Herpes simplex virus (HSV) can infect a broad host range and cause mild to life threating infections in humans. The surface glycoproteins of HSV are evolutionarily conserved and show an extraordinary ability to bind more than one receptor on the host cell surface. Following attachment, the virus fuses its lipid envelope with the host cell membrane and releases its nucleocapsid along with tegument proteins into the cytosol. With the help of tegument proteins and host cell factors, the nucleocapsid is then docked into the nuclear pore. The viral double stranded DNA is then released into the host cell’s nucleus. Released viral DNA either replicates rapidly (more commonly in non-neuronal cells) or stays latent inside the nucleus (in sensory neurons). The fusion of the viral envelope with host cell membrane is a key step. Blocking this step can prevent entry of HSV into the host cell and the subsequent interactions that ultimately lead to production of viral progeny and cell death or latency. In this review, we have discussed viral entry mechanisms including the pH-independent as well as pH-dependent endocytic entry, cell to cell spread of HSV and use of viral glycoproteins as an antiviral target.

## Introduction

Millions of people worldwide are exposed to herpes simplex virus (HSV) (Looker et al., 2015), following the exposure the virus may remain asymptomatic or may cause mild to life threatening complications (Ramchandani et al., 2016). HSV can be broadly divided into two serotypes: HSV-1 and HSV-2 (Kelly et al., 2009; Fontana et al., 2017) HSV-1 infections are primarily associated with mild to severe symptoms including blisters and inflammation of oral and ocular cells but in some cases, they can progress to more serious illnesses including blindness, hearing impairment, and fatal encephalitis (Koujah et al., 2019; Lobo et al., 2019). Similarly, HSV-2 infections may cause mild genital lesions but can also increase the risk of acquiring and transmitting fatal human immunodeficiency virus (HIV) infections (Looker et al., 2017). Additionally, both HSV-1 and HSV-2 can interchangeably infect oral or genital sites (Agelidis and Shukla, 2015).

HSV-1 and HSV-2 belong to the family Herpesviridae, all of which have unique four layers: the central double stranded DNA, enclosed by an icosapentahedral capsid, which is surrounded by a group of tegument proteins, which in turn, are encapsulated in a lipid bilayer envelope containing membraneproteins and glycoproteins (Karasneh and Shukla, 2011). The Herpesviridae family is classified into three subfamilies: alpha-herpesviruses, beta-herpesviruses, and gamma-herpesviruses subfamilies. All members of the *Herpesviridae* family establish latency (the ability of a virus to remain dormant within the host cell), but the cells in which they establish latency vary. Most Alpha-herpesviruses establish latency in neurons, beta-herpesviruses establish latency in non-neuronal cells, and gamma-herpesviruses establish latency in B and T lymphocytes (Kelly et al., 2009). But there are few exceptions, for example Marek’s disease virus is an Alpha-herpesvirus but establish it’s latency in chicken CD4+ T-cells (Parcells et al., 2003).

HSV-1 and HSV-2 belong to the alpha-herpesvirus subfamily, generally have a short replicative cycle and are capable of infecting broad host range. The mature HSV consists of the following: 1) a linear double stranded DNA of ~152 kb encoding at least 74 genes, 2) encased in an icosapentahedral capsid composed of 162 capsomeres made of six different viral proteins, 3) surrounded by 20-23 different viral tegument proteins that have structural and regulatory roles (Albecka et al., 2017), and 4) covered by an envelope that has at least 12 different glycoproteins: gB, gC, gD, gE, gG, gH, gI, gJ, gK, gL, gM, and gN on their surface, in distinct shapes and sizes. Some exist as heterodimers (gH/gL and gE/gI), while most exist as monomers. Upon exposure to a suitable host, viral glycoproteins attach to the host cell surface receptors (viral attachment). Later they interact with each other (glycoproteins) and fuse the viral envelope with the host cell membrane, thereby delivering the viral content into the host cell. The presence of four glycoproteins: gB, gD, gH, and gL and their host cell receptors has been reported to be sufficient to deliver viral content into the host cell (Karasneh and Shukla, 2011).

This review gathers and details the experimental evidence and pioneering research on the direct membrane fusion mechanism of the HSV and its essential components. As a central mechanism, the binding of four viral glycoproteins gD, gH/gL and gB to its specific receptors releases the viral contents into the cell (Karasneh and Shukla, 2011). First, the virus attaches to the host’s cell surface receptor, heparan sulfate proteoglycans (HSPGs) via its viral glycoproteins gB and/or gC (WuDunn and Spear, 1989; Herold et al., 1991; Shukla et al., 1999). The virus then slides on the cell surface and reach the cell body, a movement termed as viral surfing (Dixit et al., 2008; Oh et al., 2010; Thakkar et al., 2017). It then binds with cell membrane receptors using gD, gH/gL, and gB glycoproteins which triggers direct membrane fusion. In this review, the process of membrane fusion, structural and functional details of these four essential viral glycoproteins, and their host cell surface receptors are discussed in detail. Also, this review briefly discusses the low pH-dependent endocytic entry, the cell to cell spread of HSV and about viral glycoproteins as an antiviral target.

## Plasma Membrane Fusion

During HSV-1 or HSV-2 infection, the virus fuses its envelope with host cell membrane with the help of fusogens. Fusogens are viral encoded transmembrane fusion proteins usually present over the surface of viral envelope. In case of HSV, gB acts as a viral fusogen. A multi-protein complex involving gB, gD, gH/gL and their cognate receptors is known as the “core fusion machinery”, and together they perform the fusion reaction (Eisenberg et al., 2012; Fontana et al., 2017; Sathiyamoorthy et al., 2017; Weed et al., 2017). The fusion reaction delivers the viral nucleocapsid and tegument proteins into the host cell (**Figure 4**).

**Figure 1:**
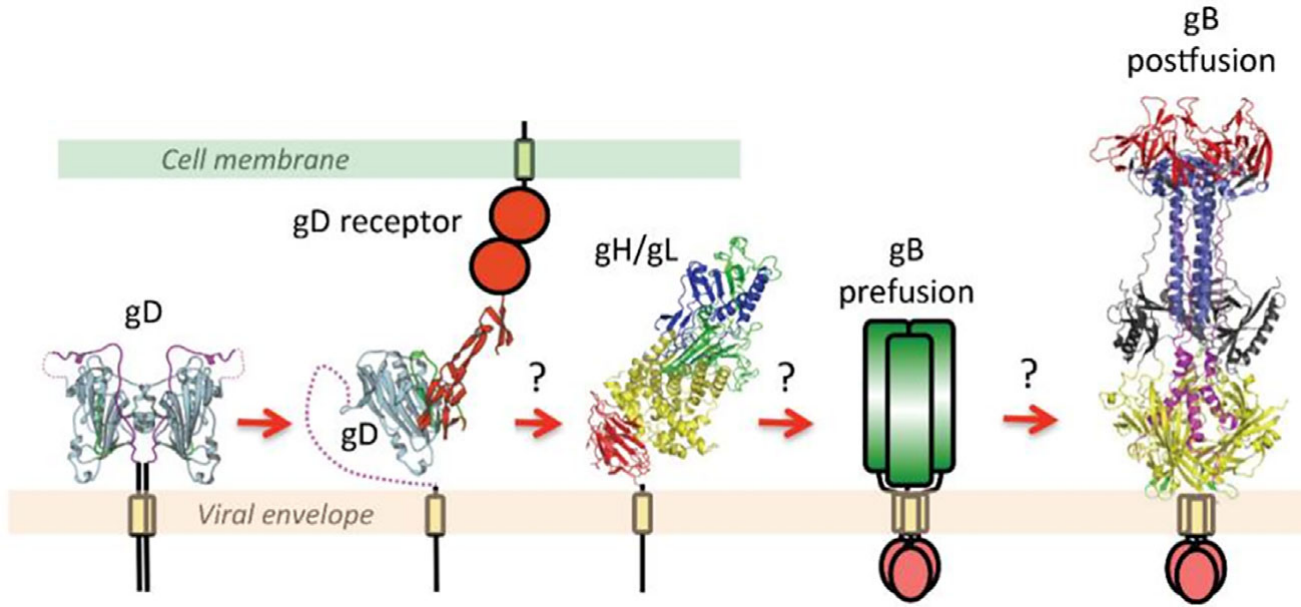
This figure is taken from the following paper published by Cooper and Heldwein in *Viruses* in 2015, “Herpesvirus gB: A Finely Tuned Fusion Machine” under the Creative Commons Attribution 4.0 International license. The original figure caption is provided as follows: “A schematic diagram of essential steps in HSV glycoprotein-mediated fusion. Crystal structures of apo gD (PDB ID 2C36), gD/HVEM complex (PDB ID 1JMA), gH/gL (PDB ID 3M1C), and the postfusion form of gB (PDB ID 2GUM) are shown. The prefusion form of gB has not yet been characterized and is shown schematically. Conformational changes in gD upon receptor binding are well documented. The order of subsequent steps has been proposed but not yet confirmed. Figure was made with Pymol.” Please note that the receptors shown are generalized and not depicting a specific protein.

According to the widely accepted model, the binding of gD to one of its cellular receptors initiates the fusion reaction (Gianni et al., 2013b). Binding causes conformational changes in the gD that changes its auto-inhibitory closed state to its active state and transmits a signal to gH/gL. These series of events activate gB by an unknown mechanism (Cooper and Heldwein, 2015; Atanasiu et al., 2016; Fontana et al., 2017); (**Figure 1**). More precisely, the gH-gL activation model proposed by the Gianni et al. in 2015 postulates gH/gL requires two signals: the first one from receptor-bound gD and the second one from the integrin (gH/gL receptor). Upon receiving these signals, gL disassociates from the complex. This allows gH to bind with its receptor and activate gB. Possibly, gL may act as an inhibitor of gH and help maintain gH in an inhibited form until it receives the appropriate signals. Thus, the disassociation of gL favors the binding of gH to its receptor and activates gH. Activated gH then transmits signals to gB (Gianni et al., 2015). Upon receiving the signal, gB undergo series of conformational changes. One study claimed that HSV gH/gL can regulate a hemifusion state of gB (Subramanian et al., 2007). However, others could not detect gH/gL mediated hemifusion (Jackson and Longnecker, 2010). In any case, the merging of membranes forms a fusion pore through which virus delivers its content into the host cell (Cooper and Heldwein, 2015; Fontana et al., 2017). In the absence of gD, gD receptors, gB, integrin or in the presence of neutralizing monoclonal antibodies to gH and gL, the dissociation of gH and gL does not take place which blocks viral fusion (Gianni et al., 2015).

Combining crystallographic structural analysis with previously published data, “the clamp and wedge model” of fusion mechanism in HSV was proposed (Cooper and Heldwein, 2015; Rogalin and Heldwein, 2015; Cooper et al., 2018). According to this model, the membrane bound cytoplasmic domain (CTD) of gB acts as a clamp and restrains the fusogenic activity of gB by stabilizing the ectodomain in a pre-fusion conformation. Upon binding to its receptor, gD undergoes the conformational change and transmits the signal to gH/gL dimer (Cooper et al., 2018). Viral gH’s ectodomain receives this signal *via* its H1 domain and transmits the signal through H2 to membrane proximal H3 domain, which then translates the signal to the cytoplasmic tail of gH. Upon receiving the message gH’s cytoplasmic tail acts as a wedge and splits the gB’s CTD clamp restrain in the cytoplasmic tail (Rogalin and Heldwein, 2015). This action releases the gB and favors the attachment of gB’s fusion loop onto the host’s surface which promotes membrane fusion. It is proposed that membrane proximal region (MPR) of the gB may contribute to lipid fusion process since this region is rich in hydrophobic amino acids. Also, the transmembrane domain (TMD) of gB may act as a conduit facilitate lipid mixing and formation of the fusion pore, ultimately leading to the release of viral content into the host cell (Cooper and Heldwein, 2015; Cooper et al., 2018).

## Viral Glycoprotein B (gB)

HSV gB is a class III fusion glycoprotein highly conserved among herpesviruses (Ruel et al., 2006; Weed et al., 2017) and least characterized (Roche et al., 2006; Roche et al., 2007; Cooper and Heldwein, 2015) gB is 904 amino-acid residues long and consists of an extended rod or spike-like ectodomain (Liu et al., 2006; Fontana et al., 2017), a hydrophobic MPR, a TMD, and a CTD. Initially, it was thought that only the gB’s ectodomain actively participates in the fusion reaction. However, recent research confirms the adjacent MPR, TMD, and CTD regions also play a key role (amino acids 730 to 904) in regulating the fusion reaction (Ruel et al., 2006; Cooper and Heldwein, 2015; Fontana et al., 2017).

**Figure 2:**
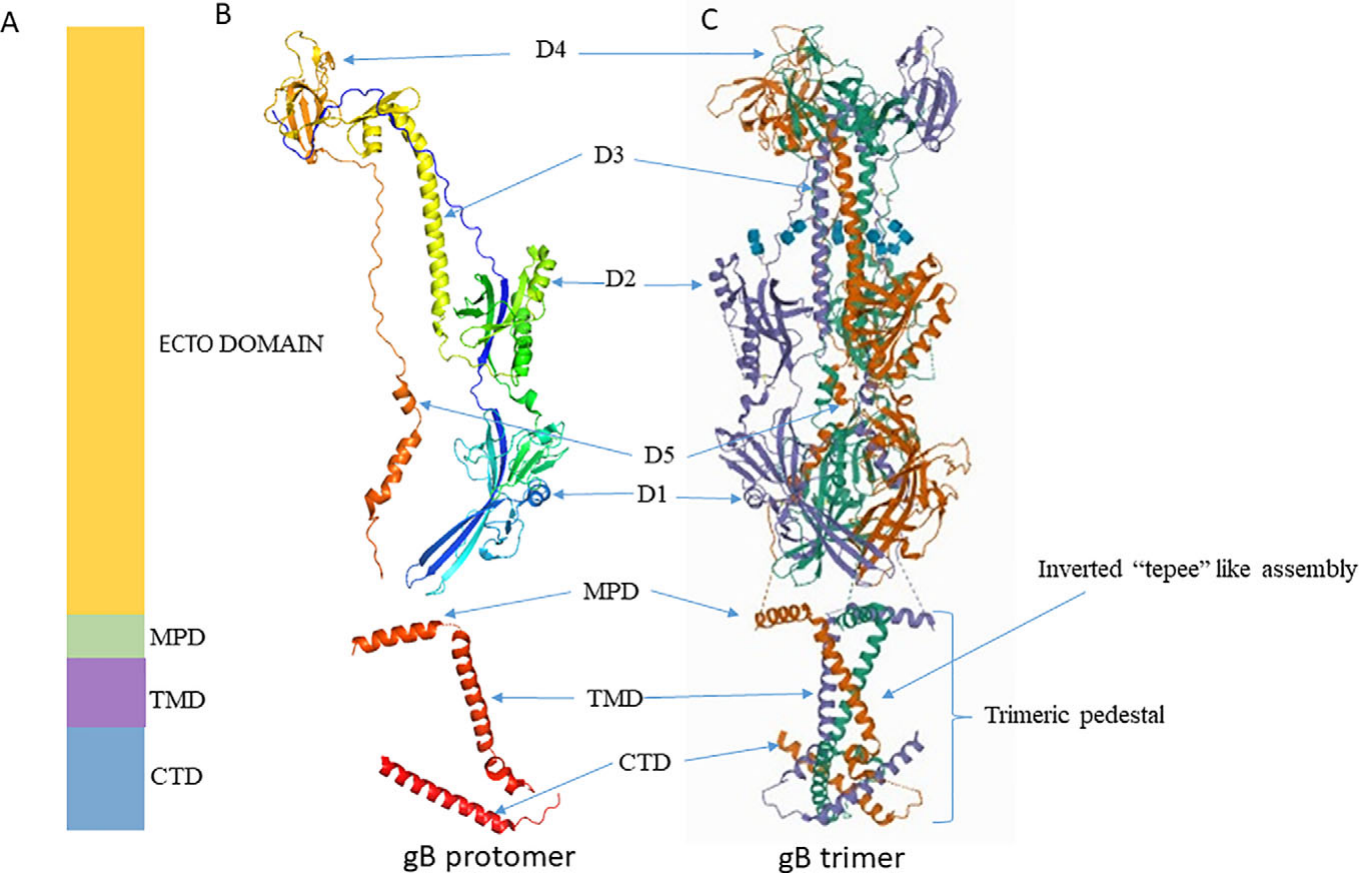
Crystal structure of gB. **(A)** Schematic representation of gB domains **(B)** Ribbon diagram of a single gB protomer (UniProtKB - P10211) **(C)** Ribbon diagram of gB trimer (PDB ID 5V2S). Figures were made with Pymol. MPD, Membrane Proximal Domain; TMD, Trans Membrane Domain; CTD, Cytoplasmic Tail Domain; D, Domain.

Crystallographic structure analysis of full-length gBΔ71 (a hybrid of a post-fusion ectodomain and the pre-fusion CTD) from HSV-1 reveals that the ectodomain in its post-fusion conformation rests upon a uniquely folded trimeric pedestal. This pedestal is composed of the MPR, TMD, and CTD, and it interacts extensively with the viral membrane (Cooper et al., 2018); (**Figure 2**). Disturbing this pedestal confirmation and it’s interaction with the membrane might be the reason why gB is always extracted in post-fusion conformation during extraction (Cooper and Heldwein, 2015). The gB’s ectodomain structure and function are greatly controlled by MPR, TMD and the CTD regions. Thus, minor changes made in these regions affect its normal structure and function. However, the mechanism by which these regions control the ectodomain is still unknown (Cooper et al., 2018).

HSV’s gB exists in two forms: pre-fusion and post-fusion form. Much of the details regarding gB’s post-fusion ectodomain is obtained from crystallographic studies while the structure and function of pre-fusion ectodomain is still unknown (Cooper and Heldwein, 2015).

### Structure of the Post-Fusion Form of gB

The X-ray crystallography structure reveals that the post-fusion form of the HSV-1 gB ectodomain appears as a trimeric (three protomers combined) spike or rod-like structure (Heldwein et al., 2006). Each protomer is organized into five distinct domains and two linker regions that forms a hairpin shape (Cooper and Heldwein, 2015; Fontana et al., 2017; Arii and Kawaguchi, 2018). These domains interact with their equivalent counterpart domain on the other protomers and form a stable trimeric structure (Cooper and Heldwein, 2015); (**Figure 2**). Domain I houses the fusion loop and is referred to as the fusion domain. Domain II mediates interactions with gH-gL, earning it the description - gH-gL binding domain (Atanasiu et al., 2010b; Cairns et al., 2013). Domain III consists of α-helices, and it forms the characteristic trimeric coiled-coil central core of the protein. Domain IV sits on top of the post-fusion form known as the crown domain and is thought to bind with cellular receptors (Karasneh and Shukla, 2011). This is supported by studies that show that the binding of antibodies to the crown domain prevents gB from binding to its cell receptor (Bender et al., 2005; Hannah et al., 2009). Lastly, domain V, known as the arm domain, consists of a long extension and connects the protomers together (Karasneh and Shukla, 2011). The flexible linker region facilitates the gB conformational change during fusion reactions (Liu et al., 2006; Lin and Spear, 2007; Karasneh and Shukla, 2011; Cooper and Heldwein, 2015). The X-ray crystallographic studies normally exclude post-fusion gB’s N terminus (amino acids 31 to 102) due to its flexibility (Fontana et al., 2017). Even though studies rationalize that gB’s N terminus does not have any unique function, it is important to note that the significance of gB’s flexible region has not been precisely identified yet.

### Structure of the Pre-Fusion Form of gB

Crystallographic studies have expanded on known information related to gB’s post-fusion form. In contrast, gB’s pre-fusion form remains relatively unclear. During extraction, the gB ectodomain directly adopts its post-fusion form, making the initial form difficult to study and examine (Vitu et al., 2013; Cooper and Heldwein, 2015). One recent study emphasizes the interaction of gB anchoring segments with its lipid bilayer in maintaining gB in its pre-fusion form (Cooper et al., 2018). Disturbing the bilayer scaffold destabilizes the pre-fusion conformation, enabling the gB to refold readily into its post-fusion conformation (Patrone et al., 2014; Cooper et al., 2018). Studies that have attempted to capture the pre-fusion form of gB by modifying it through point mutations, deletions and truncations have not been able to document the pre-fusion form (Vitu et al., 2013).

However, some have captured the 3D image of gB in its pre-fusion form by expressing full-length gB embedded in microvesicles (Zeev-Ben-Mordehai et al., 2016; Fontana et al., 2017). One of the studies have captured the image of gB’s pre-fusion form using cryo-electron tomography (cryo-ET) and subtomogram averaging with the help of a series of fusion protein-modified forms of gB and anti-gB antibodies. They propose the pre-fusion form of gB has a globular structure adjacent to the membrane and that it is approximately 8 nm tall and 7 nm wide (Fontana et al., 2017). Based on the observations, the authors suggest that conversion of gB to its post-fusion form requires series of changes to take place in its pre-fusion form. The first change occurs at domain V or at the MPR that allows fusion loops to point away from the viral membrane and toward the host membrane. This results in compacting intermediate conformation 1 which does not attach the fusion loops to the membrane surface. The second change occurs at domain III which allows gB to adopt its extended intermediate conformation 2 that attaches the fusion loop onto the surface of the host membrane. Finally, changes in domain V convert gB to its post-fusion conformation. This conformation brings the viral and host cell membranes close to each other and favors the membrane fusion (Fontana et al., 2017). However, direct experimental evidence on how gB undergoes transition from pre-fusion to post-fusion states is lacking, and the factors that contribute to these changes remain unclear.

### Membrane Proximal Region of gB

Crystallographic studies have revealed that the MPR is approximately 43 amino acid residue long and helical in structure. This region is highly hydrophobic in nature, and it is not part of the post-fusion hairpin (Shelly et al., 2012; Maurer et al., 2013). The MPR lies in between the ectodomain and the TMD, and it is seen in parallel to the membrane bilayer (Cooper and Heldwein, 2015).

The specific function of MPR region is still unknown, but studies have shown alterations like point mutations, deletions, or insertions in this region have a negative impact on viral infectivity (Rasile et al., 1993; Zheng et al., 1996; Lin and Spear, 2007; Shelly et al., 2012; Cooper and Heldwein, 2015; Efler et al., 2015). Experts believe this region determines the lipid mixing during fusion reaction. When conditions are favorable, specific amino acid residues in this region facilitate the attachment of the virus to the host cell membrane. Similarly, when conditions are unfavorable, another set of amino acids in this region shields and isolates the fusion loops, thereby preventing the fusion reaction (Shelly et al., 2012; Cooper and Heldwein, 2015). In some viruses, this region is involved in the formation of fusion pores (Li and Blissard, 2009) and is essential for cell to cell fusion (Jeetendra et al., 2003). Yet, direct experimental evidence proving the function of this region in HSV is lacking.

### Transmembrane Region of gB

The membrane-bound single pass TMD of gB is approximately 20–22 residues long (Rasile et al., 1993; Gilbert et al., 1994). TMD lies between MPR and CTD (**Figure 2**). It is helical in structure and perpendicularly positioned to the MPR helix and membrane bilayer (Engelman et al., 1986; Arkin and Brunger, 1998). Recent crystallographic study reveals single-pass TMD helices of each promoter cross one another at a 46° angle to form a unique “inverted tepee” like assembly (Cooper and Heldwein, 2015). The N terminals (proximal to MPR) are splayed, and the C terminals (proximal to CTD) are converged thus uniquely forms the inverted tepee structure (**Figure 2**). Amino acid residues in TMD are highly conserved among α-herpesviruses, implying a structural and functional importance of this region in α-herpesviruses. Experiments that replace the TMD with a lipid anchor demonstrate that HSV-1 does not proceed beyond hemi-fusion stage, which denotes that gB TMD is not just a membrane anchor but has essential roles in the later stages of fusion (Engelman et al., 1986). The MPR and TMD may not initiate fusion reaction, but once fusion is initiated by other factors, they facilitate lipid mixing and formation of fusion pore (Markosyan et al., 2000; Bissonnette et al., 2009; Cooper and Heldwein, 2015).

### Cytoplasmic Tail

Structural analyses have revealed that the 109 residue long cytoplasmic tail of HSV-1 gB is organized into domains (H1, H2 and H3) and linkers. The H1 domain further contains subdomains H1a, H1b, and a linker. The H1a and H2 domains forms α-helices, H1b forms a 310 helix, and the structure of H3 is unresolved. Similarly, the structure of the linker that connects H1b, H2, and H2, H3 is unresolved. Long H2 α-helices form the central trimeric core beneath the membrane, and they are angled such that one end faces the membrane while the other end forms a triangular base below the zigzag protrusion. The zigzag protrusion around the central core is formed by H1a and H1b along with TMD. Conserved proline residues (P805 at TMD/H1a and P811 at H1a/H1b junction) create this zigzag protrusion, and they are essential for overall structural stability (Cooper et al., 2018).

Studies that alter cytoplasmic tail domain have shown the importance of this region, as mutations, truncations and insertions in this region affect viral infectivity, especially by enhancing cell fusion (Weed et al., 2017). In cell culture, wild-type HSV does not normally form syncytia (individual cells fused to multi-nucleated cells), but an alteration in the CTD region of gB favors syncytia formation during infection (Cooper and Heldwein, 2015). This suggests that this region of gB is thought to negatively regulate fusion reactions and maintains gB in its pre-fusion form, preventing cell fusion.

As discussed earlier, experts believe the CTD of gB act as a clamp and controls the pre-mature formation of gB’s post-fusion. Upon receiving proper signal, cytoplasmic tail of gH/gL releases the clamp which frees gB to unfold to its post-fusion conformation and promotes cell fusion. The role of the CTD clamp may be unique to HSV since other herpesviruses have their own ways of controlling the pre-mature formation of gB’s post-fusion conformation (Cooper and Heldwein, 2015).

## Viral Glycoprotein gD 

HSV gD is a 369 amino acid residue long type I membrane glycoprotein that is approximately 8-10 nm long and irregularly clustered on the viral membrane surface. HSV gD is organized into an ectodomain, a TMD and a short cytoplasmic tail. Though the gD of all alpha-herpesviruses serves a similar function – binding to host cell receptor and initiating fusion reactions (Cocchi et al., 2004) – it is not replaceable. Experiments that tried replacing it have reported a complete loss of function (Fan et al., 2014; Fan et al., 2017).

According to crystallographic studies, the gD ectodomain has an immunoglobulin-like core, edged by N and C terminal extensions on either ends (Krummenacher et al., 2005; Karasneh and Shukla, 2011). The N-terminus domain is termed as the receptor binding domain (RBD), and this part of the gD binds with specific host receptors. The C-terminus domain is termed as pro-fusion domain (PFD) which interact with gH/gL and gB (Fan et al., 2017). The PFD also binds with the N-terminus region and forms an auto-inhibitory closed conformation. This self-inhibitory conformation is essential to prevent the binding of gD to gH/gL or gB before it binds to its specific receptor. The binding of gD to its specific receptor causes conformational changes in the gD that favor the release of the PFD domain from its N-terminus interaction. This release then allows PFD to bind with gH/gL or gB. Thus, self-inhibitory confirmation prevents the premature binding of gD to gH/gL and or gB. The interaction of PFD with gH/gL and gB is essential for the formation of core fusion machinery (Karasneh and Shukla, 2011). Studies have shown the binding of antibodies to this specific region, blocks the interaction of gH/gL and gB to gD. The PFD region is rich in proline residues (P261-P305) and located proximal to the transmembrane segment. Unfortunately, crystallographic studies could not resolve the structure of this region (Cocchi et al., 2004).

RBD and PFD are both essential for the fusion reaction. The infectivity of a HSV-1 lacking gD can be restored upon addition of exogenous soluble gD but only if the PFD and RBD regions of the gD ectodomain are present in that soluble form as these regions cannot function independently (Gianni et al., 2009; Fan et al., 2014; Fan et al., 2017). The RBD of gD is essential for it to recognize its receptors as well as binding of gB to its receptors, especially to PILRα (Satoh et al., 2008). A recent study that investigated the plasticity of gD by analyzing several gD mutation constructs supports this idea. PQF170 is one such mutation construct, in which residues 1–32 were deleted. When authors performed the quantitative cell fusion assay to assess the cell fusion activity of the mutants, they observed the mutant lost its binding efficiency with HVEM but retained its binding efficiency with nectin-1. Interestingly, the mutant also lost its binding efficiency with PILRα (gB receptor). This suggests the first 22 amino acids of gD may be essential for gD to recognize its receptors as well as the binding of gB to its receptor (PILRα). This result supports the idea PILRα requires gB as well as gD to induce cell fusion (Fan et al., 2009). These studies illustrate both PRD and RBD both have a crucial role in cell fusion.

Though exogenous soluble gD is enough to restore cell fusion in the presence of other glycoproteins, the MPR, TMR, and CTD of gD might play a unique role during or after the cell fusion reaction. Studies have shown that the membrane-proximal basic residues of gD induce the formation of microvillus-like projections from the plasma membrane of transfected cells (Arii et al., 2016; Carmichael et al., 2019). A mutant in the membrane-proximal basic residues prevents this formation and reduces the viral spread (Nicola, 2016). Also, an arginine/lysine cluster located at the transmembrane-cytoplasm interface of gD critically contributes to viral spread and cell to cell fusion (Nicola, 2016; Carmichael et al., 2019).

## Viral Glycoprotein gH/gL

The HSV heterodimer gH/gL is vital for the fusion reaction, but its precise role is not understood yet (Weed et al., 2017). Previous studies predicted gH may have fusogenic properties (Kinzler and Compton, 2005; Subramanian et al., 2007). In contrast, crystallographic studies conclude that gH/gL is not a viral fusogen as it does not possess any of the reported structural features (trimeric hairpin bundle or internal fusion peptides) of other fusogens (Harrison, 2008). Studies predict gH/gL may interact with gB and gD and thus regulate the fusion reaction. Supporting this concept, neutralizing antibody study shows that gB–gH–gL complex occurs prior to fusion reaction (Muggeridge, 2000). Sequence and structural data have revealed gH/gL heterodimer of both HSV-1 and HSV-2 are similar in sequence and structure. Even the antibodies specific for HSV-1 gH/gL can bind with HSV-2 gH/gL and vice versa (Jha et al., 2016). The gH of both HSV serotypes is a type I membrane glycoprotein consisting of 838 amino acids, with a large ectodomain, a single-pass transmembrane segment and a short cytoplasmic tail of 14 amino acids (Peng et al., 1998; Weed et al., 2017). gL contains 224 amino acids, lacks a transmembrane domain, and is non-covalently bound to the N-terminus of gH (Peng et al., 1998; Chowdary et al., 2010). The gH/gL heterodimer is smaller than gB but larger than gD. The gH/gL heterodimer need each other for their proper folding and structural stability (Jha et al., 2016).

### The gH/gL Ectodomain

**Figure 3:**
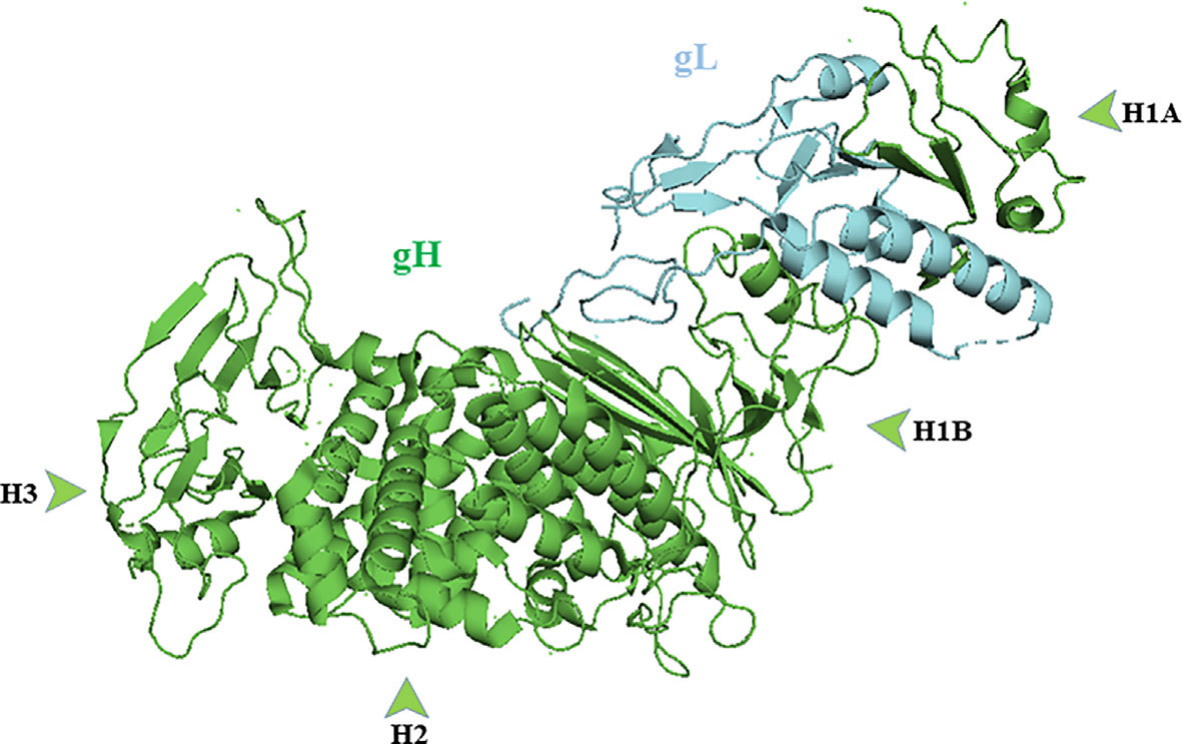
Crystal structure of gH/gL complex (PDB ID 3M1C). gH domains are in green and gL is in blue. Figure was made with Pymol.

Crystallographic studies reveal the binding of gH with gL forms a boot-shaped ectodomain that is approximately 80 Å tall and 70 Å long (Chowdary et al., 2010). The gH ectodomain is organized into three distinct domains: H1, H2, and H3 (N-terminal H1 and H2 and C- terminal H3). Sequence data suggest membrane distal domain H1 is the least conserved, H2 moderately conserved and H3 is highly conserved among herpesviruses. Domain H1 consists of subdomains H1A and H1B and a 20 amino acid residue linker that connects them (Jha et al., 2016); (**Figure 3**). gL is always seen in association H1 domain. Sequences of domain H1 and gL vary among herpesviruses and cannot be interchanged except between HSV-1 and HSV-2 (Muggeridge, 2000; Cairns et al., 2005). Subdomain H1A and H1B, hold gL like “tongs”. The interacting surfaces of H1A and gL are highly complementary as the two proteins need each other to fold properly and function normally (Hutchinson et al., 1992). The highly diverse H1 domain may receive a variety of activating signals (Cooper and Heldwein, 2015). The conserved H2 and highly conserved H3 domains then translate these diverse inputs into a common message and transmit it to gB. The conservation of domain H2 and H3 is essential for message transmission during gB activation (Cooper and Heldwein, 2015). Using monoclonal antibodies, it has been shown that gB and gD bind to gH/gL at different sites. The binding of monoclonal antibodies in that specific region prevents the interaction of these glycoproteins and formation of fusion reaction (Atanasiu et al., 2013).

### The gH Transmembrane and Cytoplasmic Tail

Studies support that full-length gH is essential to form the core fusion machinery (Browne et al., 1996; Harman et al., 2002). Full-length gH activates gB more efficiently than its soluble form (Atanasiu et al., 2010a). Replacing its cytoplasmic tail with analogous domains (Jones and Geraghty, 2004) or amino acid substitutions made within transmembrane or cytoplasmic tail did not promote cell fusion (Cooper et al., 2018) or cell to cell fusion (Harman et al., 2002). Mutational studies prove that the gH cytotail regulates and activates core fusion machinery. Insertions or truncations of the gH tail directly affect the fusogenic property of the virus. Changes either reduce or completely disturb viral infectivity or cell to cell fusion (Jackson et al., 2010; Rogalin and Heldwein, 2015; Cooper et al., 2018). The cytoplasmic tail of gH has been proposed to influence gB *via* “inside-out signaling” on the CTD region of gB (Rogalin and Heldwein, 2015). As discussed earlier, the gH cytoplasmic tail may act as a wedge which disrupts the gB clamp and promotes the fusion reaction. Thus, truncation or insertion in the tail region affect the ability of gH to reach the gB CTD and inhibits fusion efficiency (Rogalin and Heldwein, 2015).

## Herpes Simplex Virus Receptors

### gD Receptors

#### Herpesvirus Entry Mediator

HVEM is the first identified gD receptor (Montgomery et al., 1996). HVEM belongs to the tumor necrosis factor (TNF) receptor superfamily and regulates host’s immune responses (Croft, 2003). It is expressed in a wide variety of immune cells including T cells, B cells, dendritic cells, natural killer cells, macrophages, polymorphonuclear cells, and in other cell types like neurons, epithelial cells and fibroblasts (Edwards and Longnecker, 2017). T cells do not express nectin-1 since HVEM acts as the primary receptor that aids HSV entry into these cells (Spear, 2004; Agelidis and Shukla, 2015). HVEM usually binds with Ig-like ligands (CD160 and BTLA) and TNF ligands (LIGHT and LT) (Edwards and Longnecker, 2017) and regulates immune function in the host. However, certain viruses including HSV use this receptor to enter the host cell. Expressing HVEM in HSV-resistant Chinese hamster ovary (CHO) cells makes them susceptible to HSV infection (Montgomery et al., 1996). Experimental evidence suggest that HVEM along with HSV, facilitate the entry of HIV-1 into HIV-entry resistant cells (Hu et al., 2017).

A study with HSV-1 gDΔ7-15 (a HSV-1 mutant that enter host cell through nectin-1 but not HVEM) concluded that HVEM is not the primary entry receptor in the cornea (Edwards and Longnecker, 2017), suggesting nectin-1 must facilitate the initial entry of HSV on the ocular surface. The same study suggests that HVEM gets overexpressed in the corneal tissue only after the HSV infection, and HVEM-dependent induced cytokines released by macrophages are responsible for the inflammation and loss of corneal sensitivity (Edwards and Longnecker, 2017). Supporting this concept, a recent study suggests that binding of HSV-1 gD to HVEM-monocyte receptor activates NF-κB (Venuti et al., 2019). It is also known that NF-κB induces an inflammatory response in the host during viral or bacterial infection, especially by activating innate immune cells (Dorrington and Fraser, 2019).

It was proposed that gD cannot bind with both nectin-1 and HVEM simultaneously since they share a common set of binding residues (Zhang et al., 2011). The N-terminal of gD binds with HVEM (Spear, 2004) in their cysteine-rich domain 1 (CRD1) (Edwards and Longnecker, 2017). Thus, deletion of 1–32 residues from gD’s N-terminal completely or partially prevents the binding of gD with HVEM but not with nectin-1 (Spear, 2004; Fan et al., 2017). Studies suggest both HVEM and nectin-1 are vital for HSV-1 corneal infection (Krummenacher et al., 2004; Arii and Kawaguchi, 2018).

During HSV-1 infection, HVEM plays significant roles in latency and reactivation (Wang et al., 2018) (Allen et al., 2014). The Latency Associated Transcript (LAT) upregulates HVEM expression which in turn downregulates host immune responses (Allen et al., 2014). Additionally, the absence of gD and the presence of the HVEM ligands BTLA, LIGHT, or CD160 enhance viral reactivation from latency (Wang et al., 2018). Thus, blocking HVEM, LIGHT, BTLA, and CD160 impede the viral latency and reactivation (Wang et al., 2018). The role of HVEM during entry is secondary to its interaction with the HSV-1 LAT during viral latency and reactivation.

#### Nectin-1 and Nectin-2

Nectin-1 and nectin-2 are type I transmembrane glycoproteins, belonging to the immunoglobulin superfamily. Both nectin-1 and nectin-2 are expressed in a wide variety of human tissues and cell lines (Campadelli-Fiume et al., 2000). They mediate cell to cell adhesion by interacting with nectin on neighboring cells (Kwon et al., 2005). During HSV entry, gD binds the nectin-1 V-domain (Holmes et al., 2019), and this binding disturbs nectin’s neighboring cell to cell adhesion function (Zhang et al., 2011). Both HSV-1 and HSV-2 can bind to nectin-1 for entry, but HSV-2 binds with nectin-2 more efficiently (Warner et al., 1998).

The N-terminus of the gD binds with nectin, and alterations made in this region affect its receptor-binding property. Deletion of N-terminus 1-32 amino acid residues from gD does not affect its nectin-1 binding efficiency (Spear, 2004; Fan et al., 2017). However, two or more point mutations at positions 215, 222, and 223 reduce the nectin-1 binding efficiency (Manoj et al., 2004). Similarly, when insertions were made at the N-terminus of gD, the length of the insertion influenced the nectin-1 binding property of the gD (Jogger et al., 2004; Fan et al., 2017). (HSV prefers binding with nectin-1 rather than HVEM or nectin -2 (Manoj et al., 2004). HSV infection and spread were seized in neural and epithelial cells in the absence of nectin-1 (Spear, 2004; Arii and Kawaguchi, 2018). When compared with nectin-2, both HSV-1 and HSV-2 binds efficiently with HVEM (Montgomery et al., 1996). However, experimental evidence has shown that amino acid substitution made at gD’s N-terminal conserved region, can enhance the gD binding efficiency towards nectin-2 (Spear, 2004).

#### 3-O-Sulfated Heparan Sulfate Proteoglycan

HSV-1 gD, but not HSV-2 gD, binds with 3-O-sulfated heparan sulfate proteoglycan (3-OS-HS) (Shukla et al., 1999). 3-OS HS is a highly sulfated form of heparan sulfate (HS): a long linear polysaccharide (glycosaminoglycan class) chain made of disaccharides (glucosamine and glucuronic acid) which when bound to sulfate-rich, highly negative charged protein (syndecan and/or glypican) forms HSPGs (Thakkar et al., 2017; Masola et al., 2018). Sulfation of glucosamine at the 3-O position by 3-O-sulfotransferases generates 3-OS-HS. Each isoform of these enzyme generates unique 3-OS HS (Karasneh and Shukla, 2011). This adds structural diversity and structural integrity (Thakkar et al., 2017). More importantly, this makes them serve as an attachment receptor for several host proteins that regulate body functions (growth factors, chemokines, cytokines, antithrombin). The unique charge distribution on HS allows it to serve as an attachment receptor to many pathogenic viruses, including HSV, especially in the neural cells (Shukla et al., 1999; Campadelli-Fiume et al., 2000; Shukla and Spear, 2001). HSV gB and/or gC initial binding with HS (Shukla et al., 1999), is not essential for membrane fusion but promotes viral adsorption on the cell surface (Banfield et al., 1995; Laquerre et al., 1998). After the initial attachment, the virus slides down the filopodia and reaches the cell body. It then uses glycoprotein gD to bind with 3-OS HS (or other receptors) and initiates a cell fusion reaction that favors viral entry into the cell (Akhtar and Shukla, 2009; Thakkar et al., 2017). The absence of HS on cell surface reduces the HSV infection by about 100 fold (Gruenheid et al., 1993).

Addition of soluble 3-OS-HS or extrinsic expression in HSV infection-resistant cells makes them susceptible to HSV-1 infection (O’Donnell and Shukla, 2008). 3-OS-HS plays a major role in mediating HSV-1 entry in primary cultures of human corneal fibroblasts and in zebrafish (Tiwari et al., 2007a). 3-OS-HS can also regulate polykaryocyte formation (Tiwari et al., 2007b). A recent study demonstrated the presence of 3-OS-HS on mouse-derived dorsal root ganglia explants and in a single cell neuronal model. The study also captured the interaction of 3-OS-HS with HSV-1 glycoprotein B (gB) and glycoprotein D (gD) during cell entry. Furthermore, treatment of these cells with heparanase, an endoglycosidase that cleaves HS chains (Masola et al., 2018) inhibited HSV-1 entry considerably and enhanced the expression of chemokines that regulates HS (Sharthiya et al., 2017). These factors highlight the significance of HS and 3-OS-HS during attachment and entry of HSV into the host cell.

Downregulation of 3-OS-HS or competitive inhibitors of 3-OS HS significantly reduce the HSV-1 entry into the host cell (O’Donnell et al., 2010). Since cationic viral glycoprotein bind with negatively charged HS, a series of small cationic peptides (anti-HS peptides) were designed as antiviral agents. The efficiency of these peptides was tested in mouse corneal model (as prophylactic eye drops) and in human cell cultures. The test results concluded that cationic peptides could prevent the viral attachment and block the viral spread in both the models. Also this experiment emphasizes the importance of binding of viral glycoprotein to HS and 3-O-HS during HSV infection (Tiwari et al., 2011).

### gB Receptors

#### Paired Immunoglobulin-Like Type 2 Receptor-α

The paired immunoglobulin-like receptor α (PILRα) family is mainly expressed in immune cells, especially in myeloid cells: monocytes, macrophages, and dendritic cells (Satoh et al., 2008; Arii and Kawaguchi, 2018). PILRα members are important surface molecules, binding with ligands to modulate the host immune response (Lu et al., 2014). Expression of the PILRα in HSV resistant cells makes them susceptible to almost all alpha-herpesviruses, except HSV-2 (Arii et al., 2009). PILRα is the first identified gB receptor. PILRα is a paired heterodimer receptor composed of an activator and inhibitor. The inhibitory receptor binds with self-antigens like MHC molecules, and the activating receptors does not bind or recognize self-antigens. HSV binds with inhibitory receptor and delivers inhibitory signals to the host cell (Karasneh and Shukla, 2011). The presence of anti-PILRα antibodies block HSV-1 infection (Satoh et al., 2008). The binding of the gB to PILRα diverts the HSV entry route from endocytosis to direct fusion (Arii and Kawaguchi, 2018).

The binding of gB with PILRα requires several ancillary factors. Studies have shown PILRα to function as a gB receptor it requires gD and its receptor (Satoh et al., 2008; Fan et al., 2009). Also, the same study suggests that the PFD region of gD is required to facilitate the gB-PILRα mediated cell fusion reaction (Fan et al., 2017). These data suggest that the gB-PILRα mediated cell fusion reaction requires gD and it’s receptors. Mutation of O-glycosylation sites on gB (threonine-53 and threonine-480) decreases PILRα-dependent viral binding and pathogenesis (Wang et al., 2009; Arii et al., 2010b). Similarly, the presence of tryptophan-139 in PILRα is essential for it to bind with gB (Fan and Longnecker, 2010). Physiological relevance of this receptor is yet to be demonstrated in an animal model.

#### Myelin-Associated Glycoprotein

HSV-1-gB binds with myelin-associated glycoprotein (MAG) or sialic-acid-binding Ig-like lectin, present over the surface of glial cells (Arii and Kawaguchi, 2018). MAG is also a paired receptor family like PILRα, and they share 5-12% homology (Suenaga et al., 2010). In glial cells, MAG regulates myelin-axon interactions and inhibits axonal regeneration. The regulation includes myelination, initiation, and myelin integrity maintenance (Karasneh and Shukla, 2011). Both HSV-1 and varicella-zoster virus (VZV) gB bind with MAG and promote viral entry (Suenaga et al., 2010). Fortunately, MAG is not expressed in epithelial cells. Thus, MAG is not the primary receptor for the HSV. Unfortunately, during acute phase infection, HSV may utilize MAG and causes neurological disorders (Karasneh and Shukla, 2011). Future studies using gene knockout animal models will demonstrate the actual use of the receptor during infection.

#### Non-Muscle Myosin Heavy Chain IIA

HSV-1 gB also binds with non-muscle myosin heavy chain (NMHC)-IIA (Arii et al., 2010a) and can mediate viral entry into cells that express it (Karasneh and Shukla, 2011). NMHC-IIA is a subunit of non-muscle myosin IIA (NM-IIA). NM-IIA is an isoform of the NM II protein (Vicente-Manzanares et al., 2009). NM-II proteins consisting of two heavy chains, two regulatory light chains, and two essential light chains. In the host cell, NM II binds with actin and regulates normal cellular functions like cell division, adhesion, movement, migration, and contraction (Karasneh and Shukla, 2011; Agelidis and Shukla, 2015). HSV-1 gB binds with heavy chain peptides of NM II molecules and triggers viral entry into the host cell.

NMHC-IIA is ubiquitously expressed in human tissues (Agelidis and Shukla, 2015). Infectivity of HSV-1 in human promyelocytic HL60 cells is directly proportional to the expression levels of NMHC-IIA; The higher the expressions level, higher the cell susceptibility to the HSV-1 infection and vice versa (Karasneh and Shukla, 2011). Similarly, anti-NMHC-IIA antibodies have been shown to inhibit HSV-1 infection in cell lines that express NMHC-IIA. These factors support the important role of NMHC-IIA as a functional gB receptor (Arii and Kawaguchi, 2018). The research data suggest that HSV-1 gB only in the absence of PILRα, binds with NMHC-IIA. The importance of this preference is not clearly understood yet. Unlike PILRα, NMHC-IIA does not influence the HSV’s mode of entry in the cell (Arii et al., 2010a).

Studies suggest that HSV infection might take over the host system and use it to support infection and spread. For example, normally NMHC-IIA is seen only in the cytoplasm but HSV infected cells rapidly express NMHC-IIA on their surface (Arii et al., 2010a). Similarly, HSV infection can reorganize the actin cytoskeleton (Clement et al., 2006) and induce filopodia formation. While the observations are intriguing a more clear understanding of the receptor’s function in HSV entry will only be obtained *via* the use of animal models that lack NMHC-IIA gene.

### gH/gL Receptors

#### αvβ6 and αvβ8 Integrins

The αV group of integrins includes αVβ3, αVβ5, αVβ6, and αVβ8. Studies indicate that αvβ6 and αvβ8 bind gH with high affinity but at different locations (Gianni et al., 2013b). The binding of αVβ6 with gH requires the presence of the gH integrin-binding motif, Arg-Gly-Asp (RGD), while αVβ8 does not (Gianni et al., 2013b). Both αVβ6 and αVβ8 are expressed in epithelial cells but only the latter is expressed in glial and dendritic cells (Nishimura et al., 1998; Gianni et al., 2013b). The αVβ5-integrin does not bind gH/gL. Studies have concluded that αVβ3-integrin binds with the gH/gL receptor with a very low affinity and does not lead to fusion reaction. However, the binding may help HSV to enter the cell *via* acidic endosome route (Gianni et al., 2010; Gianni and Campadelli-Fiume, 2011).

Experimental evidence suggests that if integrins were blocked by antibodies or if their expression is silenced, HSV entry is restricted. In contrast, if integrins were expressed in integrin-negative cells, HSV entry is favored (Gianni et al., 2013b). Conditions like tissue remodeling (Thomas et al., 2006) and epithelial malignancies (Nishimura et al., 1998) upregulate the expression of αVβ6 in tissue. These conditions favors HSV infection (Petermann et al., 2009). It is proposed that the interaction of HSV gH/gL with integrins results in gL dissociation and is essential for activation (Gianni et al., 2015). Studies have also found that gH/gL can trigger NF-κB activation and innate immune responses through αvβ3-integrin or toll-like receptor 2 binding (Leoni et al., 2012; Gianni et al., 2013a), but the significance of these findings remains unknown.

## Low pH-Dependent, Endocytic Entry of Herpes Simplex Virus

**Figure 4:**
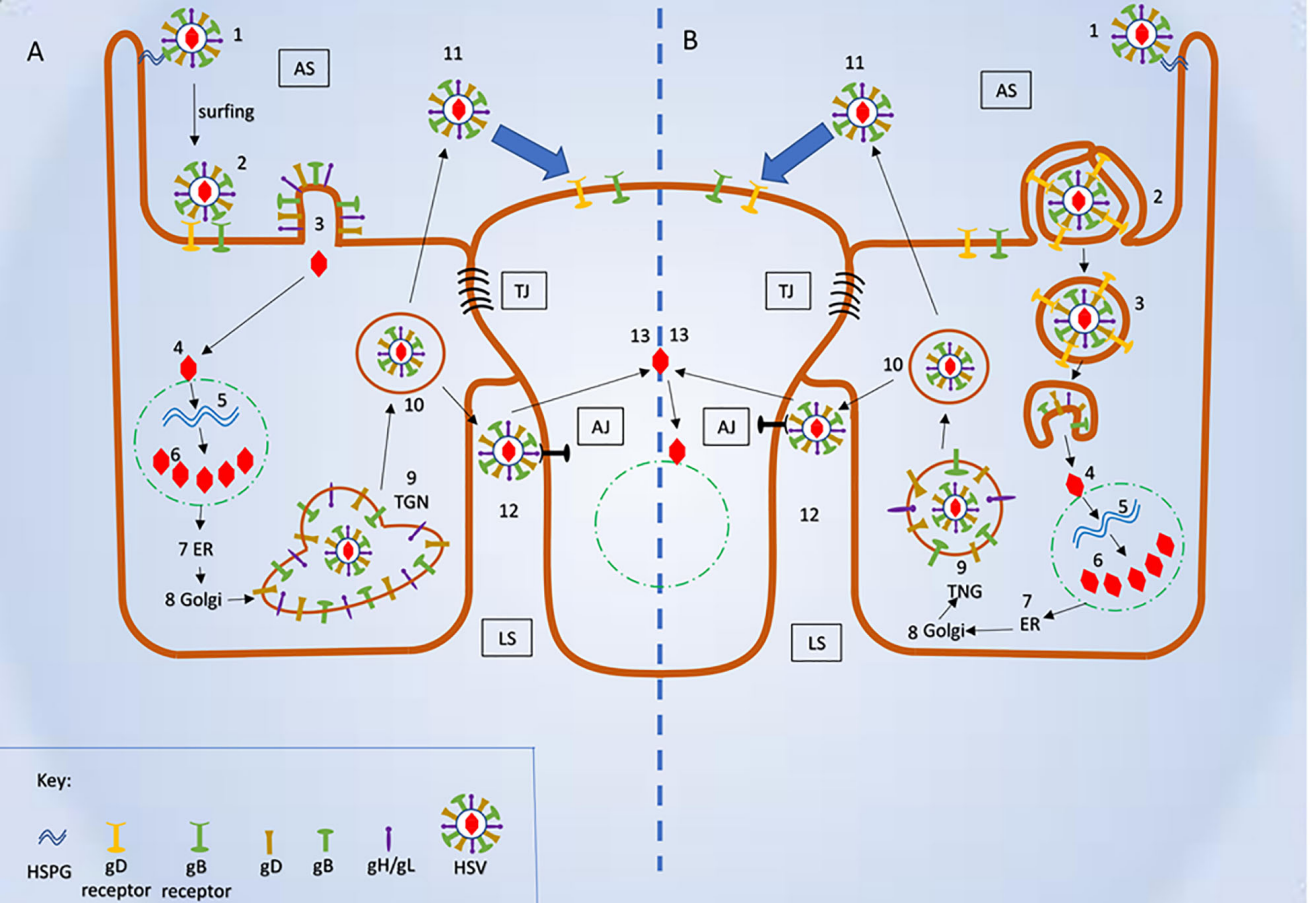
Schematic representation of herpes simplex virus (HSV) mode of entry and cell to cell spread. **(A)** Plasma membrane fusion and cell to cell spread (1) Virus attached to HSPG. (2) Attachment of viral glycoproteins with host cell receptors. (3) Fusion of viral envelope and release of viral content into the host cell’s cytosol. (4) Fusion of viral capsid and release of viral DNA into nucleus. (5-6) Replication of viral DNA and assembly of nucleocapsid. (7-9) Release of newly synthesized nucleocapsid into the Trans-Golgi network (TGN) *via* ER and Golgi. (10) Mature herpes simplex virus (HSV) inside the secretory vesicle. (11) Mature HSV released into apical surface and it is ready to infect uninfected host cell. (12) Mature HSV released into lateral surface and it infects the adjacent uninfected cell *via* binding to adhesion transmembrane proteins and release the capsid into the cytosol. **(B)** Viral entry endocytic pathway and cell- cell spread (1) Virus attached to HSPG. (2) Pseudopodia like projection engulf viral particles along with the host cell receptors. (3) Formation of endosome and fusion of viral envelope with the endosomal membrane and release of viral content into the host’s cytosol. (4) Fusion of viral capsid and release of viral DNA into nucleus. (5-6) Replication of viral DNA and assembly of nucleocapsid (7-9) Release of newly synthesized nucleocapsid into the TGN *via* ER and Golgi. (10) Mature HSV inside the secretory vesicle. (11) Mature HSV released into apical surface and it is ready to infect uninfected host cell. (12) Mature HSV released into lateral surface and it infects the adjacent uninfected cell *via* binding to adhesion transmembrane proteins and release the capsid into the cytosol. AS, Apical surface; LS, lateral surface; TJ, tight junction; AJ, Adhere junction; HSPHG, heparan sulfate proteoglycan; ER, endoplasmic reticulum; TGN, Trans Golgi network.

While this article’s main emphasis is to review pH-independent entry mechanisms, it is also important to note how HSV enters the host cell *via* a low pH-dependent, endocytic pathway. During this process, the virions are internalized and transported into the host cell’s early endosomes. The mild acidic pH of the endosome induces favorable conformational changes in the viral fusion proteins to fuse the viral envelope with the vesicular membrane (Nicola, 2016). Fusion releases the nucleocapsid from the vesicle into the cytosol, probably close to the nucleus (**Figure 4**).

Though considerable research has been done in the low pH-dependent, endocytic entry of HSV, it is still unclear what initiates the internalization. It is clear that the process is atypical endocytosis demonstrating many attributes of non-professional phagocytosis (Clement et al., 2006). This mechanism cannot be micropinocytosis since it induced only in the presence of entry receptors. Complement-mediated HSV internalization has also been reported (Van Strijp et al., 1989). Once internalized, the virus reaches the cell’s early endosomes. Mild acidic pH in endosome activates most of the viral glycoproteins (Dollery et al., 2011). In any mode of entry, the binding of gD to its host receptor is vital for its activation since mild acidic pH has no detectable effect on gD (Dollery et al., 2011). Electron microscopic and fluorescence microscopy have shown the presence of nectin-1 (or HVEM) receptor in the internalized vesicles and virus attached to the vesicles (Clement et al., 2006). Additionally, a mildly acidic pH is not a barrier to gD binding to its receptors (Dollery et al., 2011). However, gB and gH-gL, in the absence of host receptors, may be dependent on endosomal pH for the conformational changes.

The endosomal pH of approximately 6.2 to 6.4 triggers conformational changes in gB (Dollery et al., 2010; Nicola, 2016). A highly fusogenic form of gB resembles the structure of gB that has undergone low pH-triggered conformation changes (Nicola, 2016). Additionally, low pH has shown to effect the antigenic structure of gH/gL (Cairns et al., 2011). However, these alterations in favor of HSV entry have not been reported yet. In short, it is likely that the receptor-bound activated gD and pH-activated gB and gH/gL associate to form fusogenic complex that leads to fusion reaction. The complex ultimately fuse the viral envelope with the host’s vesicle and releases the viral nucleocapsid and tegument protein into the cytosol (Clement et al., 2006). Interestingly, apart from activation, a drop in pH may also serve as a “cue” for the virus to escape the endocytic pathway before it reaches lysosome (Nicola, 2016).

## Cell-to-cell Viral Spread

HSV primarily infects cells that form extensive cell to cell contact. These types of cells are called polarized cells, a group which includes epithelial cells. One of the main advantages of infecting polarized cells is that after replication, virions can move rapidly and effectively from an infected cell to adjacent uninfected cells. During this movement, they use host’s adhesion transmembrane proteins as their binding receptors, which exist to bind the host cells together. HSV moves between the cells using cell junctions adhesion proteins to avoid being detected by the host immune system (Johnson and Huber, 2002). This spread can be within the same type of cells, or it can be from the primary site to sensory site. The secondary site mostly refers to neuronal cells where they establish latency or can cause encephalitis (Johnson and Huber, 2002; Carmichael et al., 2018). The entry essential glycoproteins (gB, gD, gH-gL) and gD receptors as well as gK play important roles in cell-to-cell spread (Weed et al., 2017). Interestingly, the most commonly found gD receptor, nectin-1 is expressed at cell junctions (Campbell et al., 2017).

Studies suggest HSV uses cell junctions, specifically tight and adherens junctions, to move from infected to uninfected cells (Mateo et al., 2015). In these junctions, HSV uses the glycoproteins gE/gI to attach to its receptors that favor cell to cell spread. Mutational, ocular model, cell culture and rodent studies support the concept that gE/gI promotes cell to cell spread during HSV infection (Johnson and Huber, 2002). gE/gI is a heterodimer that may function exclusively in keratinocytes, epithelial cells, and neurons cells - the type of cells that are susceptible to HSV infection. Mutational studies demonstrate that the tail of the gE plays the primary role in delivering virions to the lateral surface of the cell. In its absence, virions are released at the apical surface (Carmichael et al., 2018) (**Figure 4**). During HSV infection, the virus accumulates glycoproteins gE/gI at the host Trans-Golgi network (TGN) and in endosomes. The glycoproteins are transferred to the envelope a process which favors lateral spread from infected to uninfected cells (Johnson and Huber, 2002; Krummenacher et al., 2003).

Apart from gE/gI, several other known and unknown viral and host factors may be involved in cell to cell spread. During cell to cell spread, HSV may induce the formation of canal-like fusion pores at the cell to cell junction which are further stabilized by the host cytoskeleton (Mateo et al., 2015). Similarly, viral glycoproteins gK, gM gN and viral tegument proteins UL11, UL16, VP22, and UL51 seem to participate in the spread mechanism (Nozawa et al., 2005; Kim et al., 2013; Wu et al., 2020). Mice infected with a gK-deleted mutant, show low cell to cell spread efficiency and relatively fewer pathological effects (Akhtar and Shukla, 2009). A recent study suggests PTP1B. a host tyrosine phosphatase, seem to be essential for the cell to cell spread. Though PTP1B modulates a wide variety of cellular functions in the host, how it aids the virus in cell to cell spread is not defined (Carmichael et al., 2018). Extracellular spread of HSV is also a route of viral transmission. Recent studies have shown that the HS cleaving enzyme, heparanse, removes the attachment receptor to facilitate viral release. This suggest that viral glycoproteins lose the ability to bind a virus-producing cell (Hadigal et al., 2015; Agelidis et al., 2017).

## Viral Glycoproteins as Antiviral Targets

Blocking HSV glycoproteins or their interaction with receptors has the potential to inhibit viral entry into the host cell, and cell to cell spread (Cai et al., 1988; Antoine et al., 2013). The interactions among gB, gD, gH-gL are essential for fusion to occur. Thus, targeting glycoprotein-receptor interactions *via* small molecules will have a significant effect in treating HSV infections. These small molecules include peptides, HS mimetics, monoclonal antibodies, aptamers, nanoparticles, synthetic, or natural compounds. A subset of those that were proven to inhibit viral entry by targeting viral glycoproteins or their receptors are discussed below.

### Peptides

Anti-HSV peptides act by interacting with viral glycoproteins or with their receptors. Most antiviral peptides are generally less toxic compared to small molecule compounds with comparative antiviral activity (Galdiero et al., 2013). However, peptides have several limitations and therapeutically limited only to topical use (Akkarawongsa et al., 2009). These peptides can be synthetic or naturally occurring. Synthetic peptides can mimic the viral glycoprotein itself. For example, synthetic peptides HB168–186, HB491514, and HB632–650 mimic the central helical region of the HSV-1 gB (Galdiero et al., 2008) and inhibit viral entry. Similarly gB122, gB131, and gB94 synthetic peptides derived from gB have been shown to restrict viral entry as well. (Akkarawongsa et al., 2009).

Synthetic peptides may lack structural similarities with viral components yet be effective in treating infections. For example, the synthetic theta defensin retrocyclin-2 inhibits HSV entry. The mechanism of action is not confirmed, but the authors speculate that it binds with gB to prevent entry (Yasin et al., 2004). Similarly, G1 and G2, 12-mer peptides that binds with HS and 3-OS HS respectively (Tiwari et al., 2011) and a 3-O-sulfated octasaccharide (Copeland et al., 2009) block the entry and spread of HSV-1 in cultured human corneal cells and in a mouse cornea model (Tiwari et al., 2011). They compete with HSV-1 for HS during attachment and effectively impede viral infection (Tiwari et al., 2011). Furthermore, the delivery of the G2 peptide through a contact lens has shown to significantly inhibit the HSV-1 entry and spread in human corneal epithelial cells (Jaishankar et al., 2016). Application of these peptides as prophylactic eye drops, have suppressed the entry and spread of HSV in *ex vivo* and *in vivo* models (Tiwari et al., 2011; Jaishankar et al., 2016). Similarly, synthetic anti-lipopolysaccharide peptides block HSV entry by binding with heparan sulfate without being toxic at the effective concentrations (Krepstakies et al., 2012).

Naturally occurring peptides can also effectively inhibit HSV entry. For example lactoferrin or it’s N-terminal domain, lactoferricin inhibits HSV entry (Jenssen, 2005). Lactoferrin, a 80 kDa peptide is part of our innate immune system and found in various secretory fluids (Antoine et al., 2013). Interestingly, preincubating human lactoferrin with HSV-1 effectively reduced viral entry and spread. However, preincubating lactoferrin with a gD-mutant HSV-1 resulted in less inhibition. This suggests that this peptide restricts viral entry by binding with viral gD or to one of its receptors (Välimaa et al., 2009). Apart from gD, lactoferrin can also interact with gC and/or HS to inhibit viral entry (Jenssen et al., 2008; Valori et al., 2008). Dermaseptins are natural peptides obtained from skin of Hylid frogs (Bartels et al., 2019). They are reported to have antiviral activity against HSV-1 and HSV-2. These peptides have shown to interact with HS and thereby interrupt the initial viral attachment to the host cell (Hadigal and Shukla, 2013). Pre-incubating HSV-2 with biochemically modified Dermaseptins have been shown to inhibit acyclovir‐resistant HSV-2 entry (Bergaoui et al., 2013). Mytilin, another entry inhibitor, is a small natural peptide obtained from the Mediterranean mussel (*mytilus galloprovincialis*). Like many of the other peptides, it inhibits viral entry by interfering with the viral attachment site on host cell receptors (Galdiero et al., 2013).

### Antibodies

Since initial studies done by Dix et al. in 1981, several studies have generated monoclonal antibodies against HSV and demonstrated their efficiency in inhibiting HSV entry into host cell. Most of these antibodies were generated against HSV glycoproteins gB, gC, gD, gH, gL, or their host cell receptor molecules (Dix et al., 1981; Nicola et al., 1998; Cairns et al., 2006; Du et al., 2017; Wang et al., 2017). Though monoclonal antibodies are effective, they are expensive, have complicated production requirements and have limited practical applications as a therapeutic agent (Chames et al., 2009).

### Aptamers and Nanoparticles

To overcome the limitations of antibodies, aptamers were created. Aptamers are short oligonucleotides that have the advantages of monoclonal antibodies such as binding target molecules with high specificity. However, they lack many of the disadvantages of antibodies. They are easy to synthesize, cost effective, less immunogenic, and 10–100-fold smaller than antibodies which allows them to penetrate tissue effectively. Aptamers may soon replace monoclonal antibodies in diagnostic and therapeutic applications (Lakhin et al., 2013). Anti-HSV aptamers are mostly specific for gD (Moore et al., 2011; Gopinath et al., 2012). Recently, we developed 45-nt-long DNA aptamers that bind to HSV-1 gD with high specificity. These DNA aptamers could significantly restrict viral entry *in vitro*, *ex vivo* and *in vivo* models (Yadavalli et al., 2017).

Our group has shown that zinc oxide tetrapod nanoparticles (ZOTEN) can inhibit viral entry as well. These micrometer-sized particles with characteristic nano-dimensional elongations trap viral particles on their surface and restrict them from entering host cells. ZOTEN, owing to its special manufacturing process, possess large amount of oxygen vacancies on their surface, giving them a strong positive surface charge. Its cationic surface charge accounts for its ability to trap virions (Antoine et al., 2016). We have also shown that ZOTEN can target and restrict both HSV-1 and HSV-2 using *in vitro*, *ex vivo*, and *in vivo* assays. Interestingly, virus-ZOTEN particles may also provide an antigen presentation platform to conceptualize a live virus vaccine (Agelidis et al., 2019).

### Heparan Sulfate Mimetics

As mentioned previously gB, gD, and gC viral glycoproteins binds HS effectively, thus molecules that mimic HS structurally have been found to inhibit HSV entry into the cell. For example sulfated heparin and its chemical derivatives (Lycke et al., 1991; Herold et al., 1995; Feyzi et al., 1997). Lignin derivative including sulfated lignins (Raghuraman et al., 2005; Raghuraman et al., 2007), and carboxylated lignins (Thakkar et al., 2010) were able to restrict HSV entry into host cells (Raghuraman et al., 2007; Thakkar et al., 2010). Several other HS mimic include pentosan polysulfate (Herold et al., 1997), dextran sulfate (Dyer et al., 1997; Herold et al., 1997), sulfated maltoheptaose (Herold et al., 1997), sulfated fucoidans (Preeprame et al., 2001; Ponce et al., 2003; Lee et al., 2004), spirulan (Mader et al., 2016), PI-88 (Nyberg et al., 2004), and nonsaccharide glycosaminoglycan mimetics sulfated galloids (Gangji et al., 2018) were successful in restricting HSV entry.

### Vaccines

Vaccines for HSV infection are still in development (Johnston et al., 2016). HSV vaccines tested in human clinical trials were primarily subunit, live attenuated, replication-defective virus based (Bernard et al., 2015). In these candidates, viral glycoproteins play a major role in stimulating the host immune response which protects the host from HSV infection (Belshe et al., 2012; Çuburu et al., 2015; Burn et al., 2018). Thus, viral glycoprotein-based subunit vaccines are seen predominantly in human clinical trials (Johnston et al., 2016). The most common glycoprotein in these vaccines is gD. Other glycoproteins such as gB, gC, gE, gK were utilized in vaccines as well (Corey et al., 1999; Stanfield et al., 2014; Awasthi et al., 2017; Egan et al., 2020). Unfortunately, some vaccines efficient in animal models failed in human clinical trials and were ceased recently. Herpevac (Belshe et al., 2012), VCL-HB01, GEN-003 (Whitley and Baines, 2018; Truong et al., 2019) were removed from human clinical trials. Fortunately, some vaccines are continuing their clinical trials. The following vaccines are currently in clinical trials or show excellent results in preclinical trials: COR-1 (Dutton et al., 2016; Chandra et al., 2019), NE-HSV2 (Truong et al., 2019), HSV-2 trivalent vaccine (Awasthi et al., 2014; Awasthi et al., 2017; Egan et al., 2020), G103 (Odegard et al., 2016), HSV529 (Bernard et al., 2015; Dropulic et al., 2017; Dropulic et al., 2019), RVX201 (Halford et al., 2011), VC2, R2 (Richards et al., 2017; Bernstein et al., 2020), HSV2 ΔgD2 (Dropulic et al., 2017; Burn et al., 2018), and HSV-2 trivalent mRNA (Egan et al., 2020). Hopefully, one or more of these may be developed into a successful vaccine to control HSV infection in the human population.

## Conclusions

The advancement of technology and contribution of scientists around the globe have unveiled several mysteries in HSV infection that were once unknown. We now understand how HSV benefits from a temporal regulation of HS whereby an abundance of HS moieties facilitates attachment and a heparanase-mediated decline in cell surface HS expression clears the way for viral egress. While using primarily pH-independent entry mechanisms, HSV has been shown to employ a pH-dependent endocytic form of entry to infiltrate the host cell. The necessity of different domains of gD to promote membrane fusion has been uncovered, and recent work has elucidated the role of cell-cell junctions during viral spread. Furthermore, many experimental antiviral therapies have emerged in recent years such as synthetic peptides, natural peptides, monoclonal antibodies, aptamers, and nanoparticles. While many of these are still being refined and investigated, they have the potential to become non-nucleoside analog therapies to treat HSV, a currently vacant niche.

However, our understanding on HSV infection is not complete yet as there are some areas where our knowledge remains cloudy. For example, the order in which its four essential viral glycoproteins are activated, their interactions, the structure of pre-fusion form of gB, and the factors initiating the internalization of HSV in endocytic pathway are unclear. After the entry of HSV into the cell, the virions neutralize almost all antiviral mechanisms of the host. Even worse, they hijack the host immune system and other signaling pathways to their benefit. Thus, stopping them before or during entry seems to be an efficient option. To do that, understanding their entry mechanisms is essential and will help us understand the host and viral factors involved in the entry of the virus into the cell and help us to prevent HSV infection in future.

## Author Contributions

KM and DS conceptualized the study. DS provided the resources. KM wrote and prepared the original draft. KM, RK, IV, TY, and DS wrote, reviewed, and edited the manuscript.RK conducted the visualization. KM, TY, and DS supervised the study. DS acquired the funding. All authors contributed to the article and approved the submitted version.

## Funding

This work was supported by the National Institutes of Health RO1 grants EY029426, AI139768, EY024710 (to DS), and an NEI core grant (EY001792).

## Conflict of Interest

The authors declare that the research was conducted in the absence of any commercial or financial relationships that could be construed as a potential conflict of interest.
